# Supplementary material for: Platelet factor 4 improves survival in a murine model of antibiotic-susceptible and methicillin-resistant Staphylococcus aureus peritonitis
Source: Front Cell Infect Microbiol. 2023 Oct 4;13:1217103. doi: 10.3389/fcimb.2023.1217103 (PMC10585365; doi:10.3389/fcimb.2023.1217103)
Supplement: Supplementary file 1 [file DataSheet_1.pdf]

SUPPLEMENTARY MATERIAL

PLATELET FACTOR 4 (PF4) IMPROVES SURVIVAL IN A MURINE MODEL OF ANTIBIOTIC-SUSCEPTIBLE AND METHICILLIN-RESISTANT STAPHYLOCOCCUS AUREUS PERITONITIS

Nataly P. Podolnikova, Valeryi K. Lishko, Robert Roberson, Zhiqian Koh, Dmitry Derkach, David Richardson, Michael Sheller and Tatiana P. Ugarova

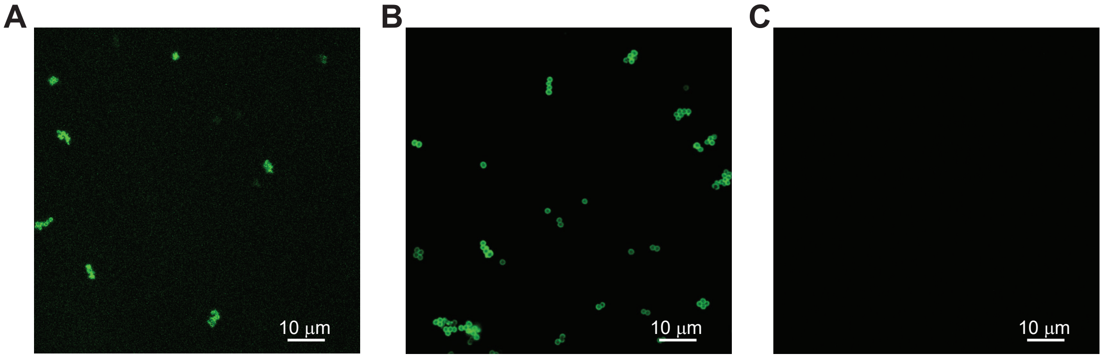

Supplemental Figure 1. Confocal images showing specific binding of anti-PF4antibody to rPF4- bound *S. aureus*. Nonencapsulated (A) and encapsulated (B) *S. aureus* was incubated with rPF4. After washing, bacteria were incubated with rabbit polyclonal anti-PF4 antibody (1:250) for 30 min at 22 ºC, followed by Alexa Fluor 488-conjugated secondary antibody, fixed and observed using confocal system. (C) Control experiment showing the lack of the secondary antibody binding to the rPF4-treated noncapsulated bacteria in the absence of the primary antibody. The scale bars are 10 µm.

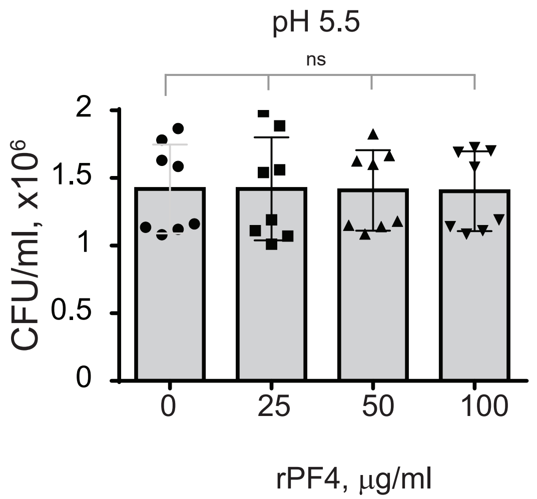

Supplemental Figure 2. Effect of rPF4 on bacterial growth. For the solution-phase microbicidal assays, *S. aureus* at 10^6 CFU/ml were inoculated into MES buffer (pH 5.5) and incubated with different concentrations of rPF4 for 1 h at 37°C. Aliquots (100 μl) of diluted suspensions (1:500) were cultured on LB agar plates. Colonies were enumerated after incubation for 24 h at 37 °C. Data are expressed as CFU/ml and are means ± S.D. from three individual experiments. ns, no significant difference.

A

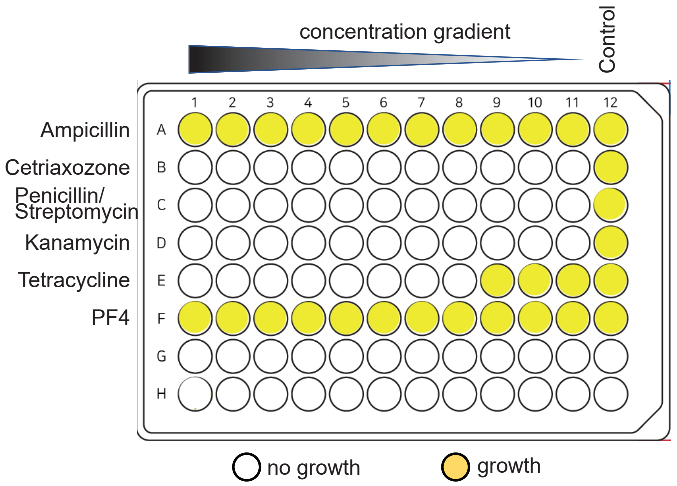

B

Antibiotic or PF4	Range of antibiotics, μg/ml	MIC, μg/ml
Ampicillin	5-5000	>5000
Ceftriaxone	0.02-20	< 0.02
Penicillin/Streptomycin	2-2500	< 2
Kanamycin	5-5000	< 5
Tetracycline	6-6000	23
PF4	0.5-500	>500

Supplemental Figure 3. Minimal Inhibitory concentration (MIC) of rPF4. (A) Aliquots of S. aureus (10^3 CFU) in LB were added to the wells containing serial dilutions (1:2) of rPF4 (0.4-500 μg/ml) and selected antibiotics. The visible growth of bacteria was evaluated after overnight incubation. Control, wells with no added inhibitors. **(B)** The concentrations of antibiotics or PF4 used in the MIC assay.

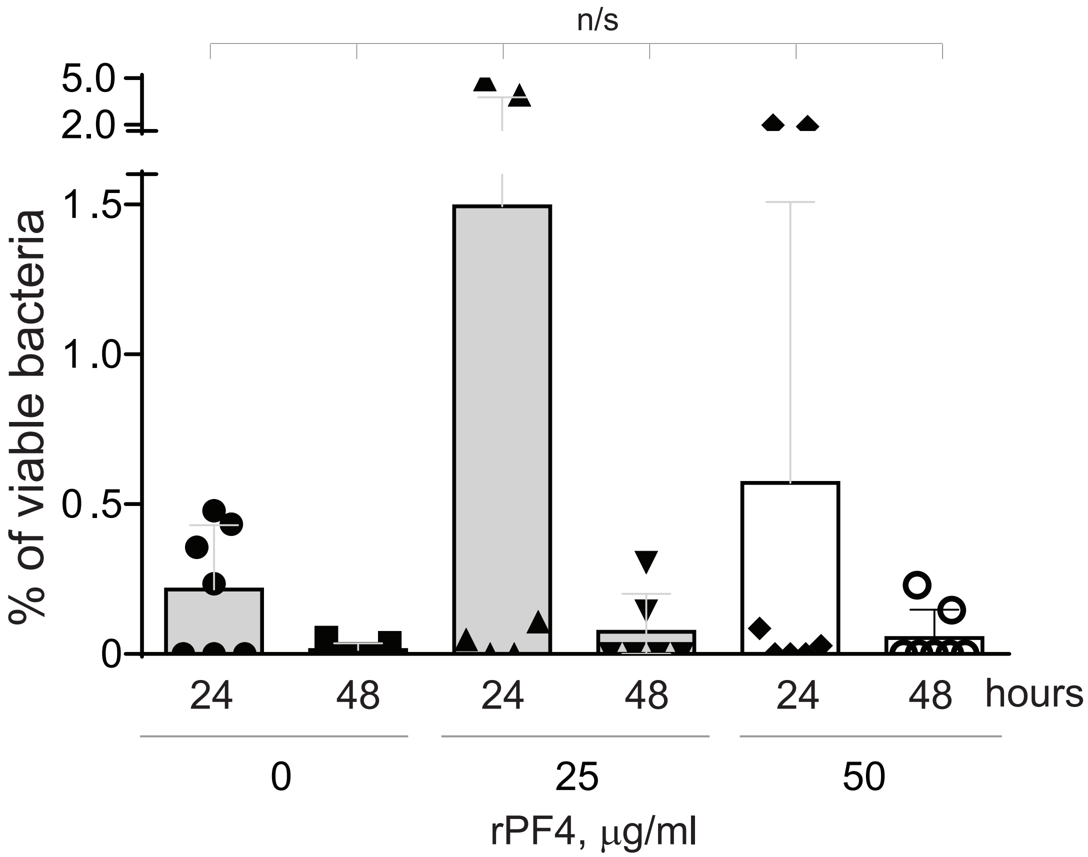

Supplemental Figure 4. Effect of rPF4 on intracellular bacterial killing. Adherent IC-21 macrophages ($5x10^5$) were incubated with 10^6 CFU of S. aureus without or with two concentrations of rPF4 (25 and 50 μg/ml) for 1 h at 37 ºC. Cells were washed, and extracellular bacteria were inactivated by treatment with gentamicin for 1 h, after which macrophages were cultured in DMEM for 24 and 48 hours and then lyzed. Aliquots of cell lysates were plated on LB agar plates for 16 h at 37 ºC, and CFUs determined. The number of viable bacteria was expressed as a percent of viable bacteria present in macrophages 1 h after gentamicin treatment. Data are means ± S.D. from six experiments.
